# The Impact of Red Blood Cell Transfusion on Blood Lactate in Non-Bleeding Critically Ill Patients—A Retrospective Cohort Study

**DOI:** 10.3390/jcm11041037

**Published:** 2022-02-17

**Authors:** Piotr F. Czempik, Dawid Gierczak, Dawid Wilczek, Łukasz J. Krzych

**Affiliations:** 1 Department of Anaesthesiology and Intensive Care, Faculty of Medical Sciences in Katowice, Medical University of Silesia, 40-752 Katowice, Poland; lkrzych@sum.edu.pl; 2 Students’ Scientific Society, Department of Anaesthesiology and Intensive Care, Faculty of Medical Sciences in Katowice, Medical University of Silesia, 40-752 Katowice, Poland; s75035@365.sum.edu.pl (D.G.); s76509@365.sum.edu.pl (D.W.)

**Keywords:** blood lactate, red blood cell, transfusion, trigger

## Abstract

Anemia should preferably be managed without red blood cell transfusion (RBCT); instead, therapy should be focused on causes of anemia along with efforts to minimize blood loss. Lactate could potentially be used as a physiologic RBCT trigger, although there are some limitations to its interpretation. The aim of our study was to analyze the impact of RBCT on blood lactate with consideration of factors known to increase its concentration and to assess the usefulness of blood lactate as a potential physiologic RBCT trigger. We performed a retrospective analysis of all RBCT episodes in non-bleeding critically ill patients. We retrieved demographic data, data on RBCT itself (duration, type of RBC, volume of RBC, age of RBC), laboratory parameters (lactate, hemoglobin, glucose, total bilirubin), and factors potentially increasing lactate. We analyzed 77 RBCTs with elevated pre-RBCT lactate. The median age of patients was 66 (IQR 57–73) years and the distribution of sexes was even. The named factors potentially influencing lactate had no impact on its concentration. The median pre-post RBCT lactate was 2.44 (IQR 2.08–3.27) and 2.13 (IQR 1.75–2.88) mmol/L, respectively (*p* < 0.01); the median decrease was 0.41 (IQR 0.07–0.92) mmol/L. We conclude that RBCT did not normalize mildly elevated lactate. Common causes of elevated lactate probably had no impact on its concentration. Therefore lactate may have a limited role as a physiologic RBCT trigger in non-bleeding severely anemic critically ill patients.

## 1. Introduction

Red blood cell transfusion (RBCT) represents the most frequently performed allogeneic tissue transplantation [1]. RBCT may be occasionally associated with acute lung injury, circulatory overload, immunosuppression, and pathogen transmission, and hence should be avoided in anemic critically ill patients [1]. Anemia should preferably be managed without RBCT; therapy focused on causes of anemia and efforts to minimize patient blood loss should be implemented, according to patient blood management (PBM). On the other, hand anemia may lead to, among other things, prolonged weaning from respirator, acute kidney injury, and myocardial ischemia [2].

It is recommended that RBCT decision making (TDM) be based on hemoglobin (Hb) concentration and clinical symptoms [3,4]. Tolerance of anemia depends on the fine balance between global oxygen (O_2_) delivery (DO_2_) and global oxygen consumption (VO_2_). Even elderly patients with chronic heart failure may tolerate very low Hb concentration [5]. RBCT is used to increase DO_2_ and improve the DO_2_-VO_2_ balance. Surrogates that reflect the DO_2_-VO_2_ balance cover but are not limited to: Arterial-venous O_2_ difference (A-V O_2diff_), O_2_ extraction ratio (O_2_ER), mixed venous O_2_ saturation, central venous O_2_ saturation (ScvO_2_), and lactate concentration. These surrogates may be used as physiologic RBCT triggers. A-V O_2diff_ was shown to be a moderate independent predictor of 90-day mortality in transfused and non-transfused patients [6]. With stable cardiac output (CO) and arterial saturation (SaO_2_), O_2_ER is calculated according to the equation O_2_ER = (SaO_2_ − SvO_2_)/SaO_2_. ScvO_2_ corresponds to SvO_2_ if Hb is fully saturated with O_2_. When myocardial O_2_ consumption cannot be ignored, there is no agreement between ScvO_2_ and SvO_2_ [7]. ScvO_2_ was described as a physiologic transfusion trigger in 2009 [8]. All these surrogates, with the exception of blood lactate, require cannulation of a pulmonary artery or a central vein in the upper body for blood sampling. In contrast to an arterial cannula which is present in almost all patients hospitalized in the intensive care unit (ICU), blood lactate is the most widely available surrogate of the DO_2_-VO_2_ balance. Lactate could potentially be used as a physiologic RBCT trigger, although there are some limitations to its interpretation. Lactate interpretation may be particularly difficult in septic patients. Change in metabolic pathways in sepsis may lead to increased lactate [9]. Pharmacological agents commonly used in the intensive care unit (ICU) may, like β-2-adrenergic receptor agonists or epinephrine, also increase lactate concentration. Regional tissue ischemia may lead to elevated lactate. Clearance of lactate may be impaired in liver dysfunction. Hyperglycemia stimulates glucose conversion into lactate [10].

The aim of our study was to analyze the impact of RBCT on blood lactate with consideration of factors known to increase its concentration and to assess the usefulness of blood lactate as a potential physiologic RBCT trigger.

## 2. Materials and Methods

**Figure 1:**
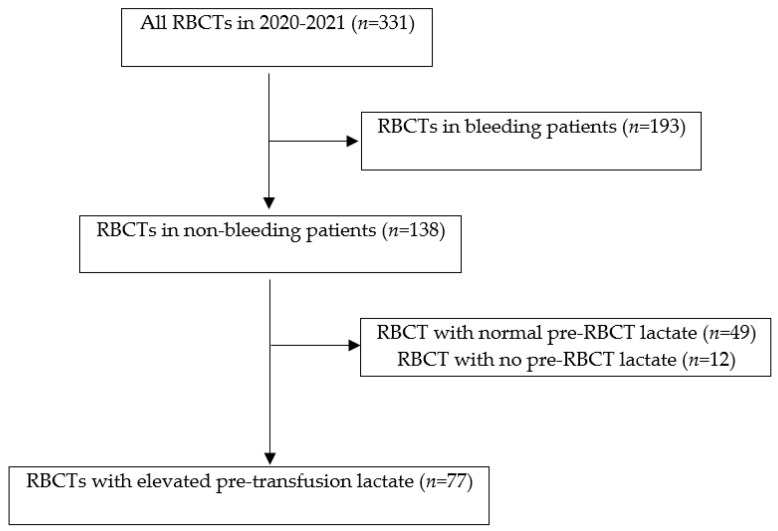
Patient inclusion based on exclusion criteria. RBCT—red blood cell transfusion.

We performed a retrospective analysis of all RBCT episodes in a cohort of non-bleeding critically ill patients hospitalized in a mixed medical-surgical ICU of a university affiliated medical center between January 2020 and December 2021. We excluded RBCTs in bleeding patients (*n* = 193). In order to determine the effect of RBCT on lactate concentration we than excluded RBCTs with normal pre-RBCT lactate concentration (49 (35.5%)) or without pre-RBCT lactate determination (12 (8.7%)). There were 77 (55.8%) RBCTs with elevated pre-RBCT lactate concentration (≥1.8 mmol/L). While the blood gas analyzer that was used in our study reported results below 1.8 mmol/L, this value is generally the detection limit for lactate in a blood gas sample. The study flow chart is presented in Figure 1. 

All data were retrieved from a transfusion book (paper version), drug charts (electronic version) and electronic health records (AMMS, Asseco, Poland). The following information on RBCT was retrieved from the departmental transfusion book: Date of transfusion, start time of transfusion, end time of transfusion, type and volume of RBC transfused, age of RBC transfused. From electronic drug charts we retrieved information on medications used on the day of RBCT. We also recorded use of medications frequently used in the ICU that may potentially increase lactate concentration, namely β-2-adrenergic receptor agonists and epinephrine [11]. From electronic health records we retrieved basic demographic parameters (age, sex) and factors frequently encountered in the ICU, which may have an impact on lactate concentration, namely sepsis/septic shock [12], liver dysfunction (total bilirubin > 1.2 mg/dL), hyperglycemia (glucose > 180 mg/dL), regional tissue ischemia (e.g., bowel ischemia, extremity ischemia), and laboratory results (hemoglobin, white blood cell, hematocrit, platelets, pH, partial pressure of CO_2_, partial pressure of O_2_, bicarbonate, lactate, glucose, total bilirubin, creatinine, blood urea nitrogen, sodium, potassium). We characterized the severity of illness in the study population with three widely used classification systems. For lactate and glucose determination, an arterial blood gas (ABG) sample (BD A-Line™ 1 mL, Beckton Dickinson, Franklin Lakes, NJ, USA) was collected. ABG was analyzed with a blood gas analyzer (RAPIDPoint^®^ 500, Siemens Healthcare, Erlangen, Germany). The reference range for lactate concentration was according to the blood gas analyzer manufacturer (<1.8 mmol/L). To minimize the risk of pre-analytical error and obtain the most accurate results, hemoglobin (Hb) was determined using a standard EDTA test tube (BD Vacutainer K2EDTA 2 mL, Beckton Dickinson). These laboratory parameters were determined in the morning (blood samples collected at 6 a.m.) on the day of RBCT. The decision regarding RBCT was made by an attending physician. There is no protocol or decision algorithm for RBCT in the local ICU; however, the restrictive approach to RBCT is usually adopted. Post-RBCT ABG was collected in the late afternoon (4–5 p.m.) on the day of RBCT. Due to lactate concentration kinetics [13], we recoded the time from the end of RBCT to collection of an ABG sample for lactate determination. Blood samples for Hb determination were collected in the morning (6 a.m.) on the next day post-RBCT.

Statistical analysis was performed using MedCalc v.18 statistical software (MedCalc Software, Ostend, Belgium). The continuous variables were presented as mean ± standard deviation (SD) or median and interquartile range (IQR). The categorical variables were expressed as numbers and percentages. We verified the type of distribution using the Shapiro–Wilk test. We analyzed intergroup differences using the Mann–Whitney test. The variables pre-post RBCT were analyzed using the Wilcoxson test. *p* < 0.05 was considered statistically significant.

Due to the retrospective and observational nature of the study, there was no requirement for a local bioethics committee approval (PCN/CBN/0022/KB/292/21).

## 3. Results

There were 138 RBCTs in 193 non-bleeding critically ill patients. We excluded RBCT with normal pre-RBCT lactate concentration (*n* = 49) and without lactate concentration determination (*n* = 12). We then analyzed 77 RBCTs with elevated (≥1.8 mmol/L) pre-RBCT lactate concentration. The median age of patients with elevated pre-RBCT lactate was 66 (IQR 57–73) years and the distribution of sexes was even. The most frequent admission diagnosis in our population was acute respiratory failure. The study population characteristics are presented in Table 1. 

For all patients with elevated pre-RBCT lactate, the median number of RBC transfused was 2 (IQR 1–2) units, the median volume of RBC transfused was 300 (IQR 250–300) mL, and the median age of RBC transfused was 17 (IQR 10–22.2) days. The types of RBC transfused were as follows: Leucodepleted 63 (82%), without buffy coat 10 (13%), and leucodepleted irradiated 4 (5%). The median time of RBCT was 30 (IQR 30–40) min and the median time from the end of RBCT and the time of blood collection for lactate determination was 3.9 (IQR 2.7–6.1) h. The median pre-post RBCT Hb concentration was 6.7 (IQR 6.35–7.05) and 7.7 (IQR 7.15–8.05) g/dL, respectively (*p* < 0.01); the median increase in Hb concentration was 1.0 (IQR 0.38–1.33) g/dL.

We analyzed intergroup differences between patients with/without named factors potentially affecting lactate and its pre-RBCT concentration (Table 2). The median value for total bilirubin in patients with liver dysfunction was 2.6 (IQR 1.94–11.47) mg/dL.

**Figure 2:**
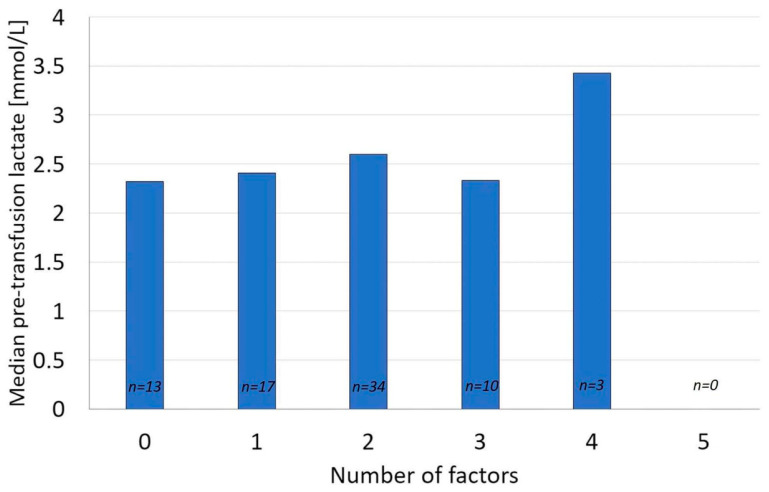
Intergroup differences in pre-transfusion lactate concentration between groups of patients with 0–5 factors potentially affecting lactate concentration (*n*–number of patients).

In 77 transfusions with elevated pre-RBCT lactate, the named factors potentially influencing lactate had no impact on its concentration in a univariate analysis. There were no statistically significant differences in pre-RBCT lactate between groups of patients with 0–5 factors potentially affecting lactate concentration (*p* 0.13–0.82) (Figure 2).

The median pre-post RBCT lactate was 2.44 (IQR 2.08–3.27) and 2.13 (IQR 1.75–2.88) mmol/L, respectively (*p* < 0.01); the median drop in lactate concentration was 0.41 (IQR 0.07–0.92) mmol/L.

## 4. Discussion

Blood lactate is a widely available surrogate of the DO_2_-VO_2_ balance [14] and may potentially be used as a so-called physiologic transfusion trigger. However, the interpretation of lactate levels has several limitations in the ICU patients. Apart from severe anaemia (Hb < 8 g/dL), there may be other clinical conditions or pharmacological agents present that may potentially increase lactate concentration [9,10]. In our study we showed that factors potentially having impact on lactate concentration that are frequently present in the critically ill patients probably had no impact on lactate concentration.

In our study we analysed only RBCT with elevated (≥1.8 mmol/L) pre-transfusion lactate in order to show the effect of RBCT on its concentration. Normal (<1.8 mmol/L) lactate does not decrease further following RBCT, as was shown in the study by Themelin et al. [15]. Themelin showed that even with normal pre-RBCT lactate, ScvO_2_ improved significantly following RBCT, showing that lactate does not have appropriate sensitivity as a physiologic RBCT trigger. In our study there were 49 RBCTs with normal pre-transfusion lactate that were excluded; in these transfusions DO_2_ still may have been improved.

In our study RBCT did lead to a statistically significant drop in lactate; however RBCT did not lead to normalisation of its mildly elevated concentration. It is impossible to know if this was due to the presence of other factors affecting lactate, or RBCT was not required and RBC over-transfusion occurred. Apart from Hb concentration, there are other factors that influence DO_2_-VO_2_ balance and lactate as its surrogate: Cardiac output, oxygen saturation, amount of oxygen dissolved in plasma, and oxygen consumption. It may also be that Hb did not reach critical levels for tissue hypoxia to occur and elevated pre-RBCT lactate was due to other factors or there was insufficient time for lactate to increase when a critical level of Hb was reached. Our observations are similar to the analysis by Mazza et al., who showed that, in the subgroup of patients with systemic inflammatory response syndrome/sepsis with Hb < 8 g/dL (mean pre-RBCT Hb 7.34 ± 0.48 g/dL), RBCT did not normalise lactate (pre-post RBCT lactate 2.02 ± 1.20 vs. 1.92 ± 0.80, *p* = 0.80) [16]. In our study pre-RBCT lactate was higher (2.46 (IQR 2.09–3.21) vs. 2.02 ± 1.20 mmol/L) and Hb was lower (6.7 (IQR 6.3–7.05) vs. 7.3 g/dL) compared to the study by Mazza et al. and still there was no normalisation of lactate post-transfusion. One should keep in mind that RBCs stored for longer time accumulate lactate as a storage lesion by-product and may impact on post-RBCT lactate levels. In our study pre-transfusion lactate was mildly elevated and lactate determination post-transfusion was performed after 3.9 (IQR 2.7–6.1) h, so lactate accumulated in RBC as a storage by-product had minimal impact on the results obtained. As RBCT did not normalise lactate even in patients with no limitations of its interpretation, we hypothesise that pre-transfusion lactate levels should not be used as a physiologic RBCT trigger in this group of patients. There are other surrogates of DO_2_-VO_2_ balance that may be used as physiologic transfusion triggers, namely ScvO_2_ [8] and A-V O_2diff_ [6].

### Study Limitations

Our study has some limitations. Firstly, it is retrospective in nature, so we missed some results, especially some pre-post RBCT lactate concentration (12 RBCTs). Secondly, the number of analysed transfusions may be a limitation; however, the number of analysed RBCT episodes had to be reduced by RBCTs with normal pre-transfusion lactate (49 RBCT), leading to a final number of analysed RBCT of 77. Thirdly, we performed our analysis in a heterogeneous group of critically ill patients. The only common factor was hospitalisation in the ICU, absence of bleeding, and severe anaemia. Moreover, the study performed was a single center study. Finally, we did not analyse other surrogates of DO_2_-VO_2_ balance, so we could not compare the validity of blood lactate to other physiologic RBCT triggers.

## 5. Conclusions

RBCT does not normalise mildly elevated lactate concentration in non-bleeding severely anaemic critically ill patients. Common causes of elevated lactate probably did not impact on lactate concentration in our group of critically ill patients. Therefore blood lactate may have a limited role as a physiologic RBCT trigger in non-bleeding severely anaemic critically ill patients. 

## Figures and Tables

**Table 1:** The study population characteristics.

Characteristic	Value
Age (years)	66 (IQR ^1^ 57–73)
Sex (male/female) (*n*)	20/20
SAPS II ^2^ (points)	49 (IQR 39–63)
APACHE II ^3^ (points)	20.5 (IQR 15.5–24.5)
SOFA ^4^ (points)	10 (IQR 6.5–12)

^1^ Interquartile range; ^2^ Simplified Acute Physiology Scale; ^3^ Acute Physiology and Chronic Health Evaluation; ^4^ Sequential Organ Failure Assessment.

**Table 2:** Intergroup differences in pre-transfusion lactate concentration in patients with and without factors potentially affecting lactate concentration. IQR—interquartile range.

Factor	No. of Patients with/without Factor	Pre-Transfusion Lactate (mmol/L)	*p*
sepsis/septic shock	Yes (*n* = 59)	2.47 (IQR 2.07–3.11)	0.47
	No (*n* = 18)	2.42 (IQR 2.23–3.53)	
Liver dysfunction	Yes (*n* = 27)	2.58 (IQR 2.12–2.32)	0.37
	No (*n* = 50)	2.42 (IQR 2.08–3.16)	
β-2-adrenergic receptor agonist	Yes (*n* = 22)	2.37 (IQR 2.05–3.15)	0.45
	No (*n* = 55)	2.48 (IQR 2.13–3.24)	
Hyperglycemia	Yes (*n* = 10)	2.71 (IQR 2.18–3.81)	0.59
	No (*n* = 67)	2.44 (IQR 2.1–3.15)	
Epinephrine infusion	Yes (*n* = 14)	3.19 (IQR 2.45–4.33)	0.06
	No (*n* = 63)	2.42 (IQR 2.09–3.01)	

## Data Availability

The data presented in this study are available on request from the corresponding author.
